# Non-coding RNA fragments account for the majority of annotated piRNAs expressed in somatic non-gonadal tissues

**DOI:** 10.1038/s42003-017-0001-7

**Published:** 2018-01-22

**Authors:** Juan Pablo Tosar, Carlos Rovira, Alfonso Cayota

**Affiliations:** 1 Nuclear Research Center, Faculty of Science, Universidad de la República, Iguá 4225, Montevideo, 11400 Uruguay; 2 Functional Genomics Unit, Institut Pasteur de Montevideo, Mataojo 2020, Montevideo, 11400 Uruguay; 3 Division of Oncology, Department of Clinical Sciences, Lund University Cancer Center, Lund, 223 81 Sweden; 4 Department of Medicine, Faculty of Medicine, Universidad de la República, Av. Italia s/n, Montevideo, 11600 Uruguay

## Abstract

PIWI-interacting RNAs (piRNAs) are regarded as the guardians of the genome because they tackle genome stability-threatening transposable elements in the germline. Recently, piRNAs were also reported in other types of cells, including mouse brain, malignant and non-malignant somatic tissues, and human plasma. This suggests that piRNA function might be broader than previously expected. Here, we show that different piRNA databases contain a subset of sequences that correspond to piRNA-sized fragments of ncRNAs (rRNAs, tRNAs, YRNAs, snRNAs, and snoRNAs) and intermediates of miRNA biogenesis. We discuss that the biogenesis of these sequences is probably independent of the PIWI pathway, and can therefore be considered contaminants in piRNA databases. Although a minority of annotated piRNAs falls in this category, they account for the vast majority of piRNA expression in somatic non-gonadal tissues. Since ncRNA fragments are ubiquitous and abundant, their confusion with piRNAs strongly impacts the estimation of piRNA expression outside of mammalian gonads.

## Abstract (web summary)

Juan Pablo Tosar *et al.* analyze existing databases to investigate whether piRNAs reportedly expressed outside the mammalian gonad are true piRNAs or likely contaminants. They conclude that the majority are fragments of non-coding RNAs from other small RNA classes rather than true piRNAs.

## Introduction

PIWI-interacting RNAs (piRNAs) are one of the three main classes of regulatory small RNAs, together with small interfering RNAs (siRNAs) and microRNAs (miRNAs). These classes differ in their biogenesis and mode of target regulation, but share some common features such as their ability to guide Argonaute proteins to target nucleic acids in a sequence-dependent manner^1^. Argonaute proteins are phylogenetically subdivided into two subclasses, comprising the orthologs of *Arabidopsis* AGO1 and *Drosophila* Piwi (defining AGO and PIWI subfamilies, respectively)^2^. While the former are involved in post-transcriptional gene silencing by siRNAs and miRNAs, the biological function of PIWI proteins was initially unclear, although they were shown early on to be essential for germ cell maintenance^3, 4^. In 2006, various groups simultaneously reported that murine PIWI proteins MIWI^5, 6^ and MILI^7^ bound a novel class of small (26–31 nt) RNAs in the testes, which they termed piRNAs. These piRNAs were encoded in discrete genomic clusters, many of which were present in syntenic genomic regions in humans.

In parallel, *Drosophila* PIWI proteins were shown to bind repeat-associated siRNAs, a germline-enriched 24–29 nt small RNA family previously known to be involved in transposon silencing^8^, in the *Drosophila* ovary. This led to the notion that the conserved function of piRNAs is to tackle genome stability-threatening transposable elements in the germline^9–11^. Mutations are particularly problematic when affecting germinal cells, and generalized demethylation of genomic DNA upon fertilization in mammals can unleash transposon expression and propagation. To avoid this, a piRNA-based innate immune system operates in the germline, comprising both genetically encoded (primary piRNAs derived from RNA pol II transcription from piRNA clusters) and adaptive (secondary piRNAs produced by ping-pong amplification) resistance mechanisms^12^.

Given the involvement of the piRNA pathway in the germline, it is not surprising that piRNAs were initially cloned and sequenced in mouse testis or *Drosophila* ovaries. However, a role for PIWI proteins and piRNAs in somatic cells has also been documented^13^. PIWI/piRNA expression was reported in larval salivary glands^14^, in the central nervous system of mice^15^ and *Aplysia*^16^. The role of piRNAs in tumors is also under study, since expression of PIWI-clade proteins was reported in many types of somatic cancer cells^13^. Indeed, a recent analysis of transcriptomic data from The Cancer Genome Atlas identified a variety of somatic piRNAs, which can distinguish tumors from non-malignant tissues^17^. Recently, more than a hundred piRNAs were sequenced in normal human plasma, and some of these were detected at high levels in every sequenced individual^18^.

If piRNAs are expressed in non-germline tissues and are even transported in the bloodstream, one could wonder whether the germline model still stands as the unique environment to study piRNA biology or whether these non-germline piRNAs are true members of this gene family. Recently, sperm-derived tRNA halves from tRNA-Gly were reported to control gene expression in early embryos^19, 20^. Interestingly, the sequences of these tRNA halves are nearly identical, with only one nucleotide variation in sequence length, to annotated piRNAs (NCBI accession: DQ597916.1 and DQ570956.1).

This observation led us to study the degree of similarity between piRNAs and ncRNA fragments. To our surprise, we found that a considerable number of human sequences in distinct piRNA databases showed 100% identity to other ncRNAs, and that these ambiguous sequences accounted for the vast majority of the piRNAs described in the mouse brain^15^, somatic cancer^17^, and blood^18^. Furthermore, these sequences do not share hallmarks of PIWI-dependent selection, such as a bias toward uridine at the 5′ end. We also show that the evidence for PIWI association to these ncRNA fragments is scarce in humans. Overall, we suggest that piRNA expression in mammalian non-gonadal cells is greatly overestimated or directly artifactual, as reported non-gonadal piRNAs are probably not bona fide piRNAs.

## Results

### Overlap between piRNA databases and non-coding RNAs

**Fig. 1:**
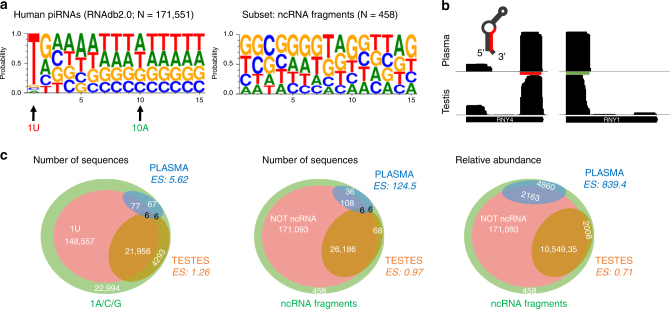
piRNAs in human plasma are non-coding RNA fragments. **a** Sequence logo showing 1 U bias in piRNA sequences in the RNAdb 2.0 database, and absence of such signature in the subset of sequences matching other known ncRNAs. For simplicity, only the first 15 bases are shown. **b** Read coverage of YRNAs (RNY4 and RNY1) in human plasma (SRR2496797) and human testis (ERA246774). The red and green bars represent the most and second-most abundant piRNAs found in human plasma in Freedman *et al*.^18^. The predicted secondary structure of RNY4 is shown, with the RNY4-derived piRNA highlighted in red. **c** Venn diagrams showing the 171,551 piRNAs in RNAdb 2.0, classified by either their 5′ start base or their identity to other ncRNAs. The piRNAs described in human plasma (blue) and human testis (orange) are overimposed, and enrichment scores (ES) were calculated as described in methods

RNAdb 2.0 and piRBase are two compendiums of piRNA sequences extracted from the scientific literature, and currently contain 171,551 and 32,826 human piRNAs, respectively. Analysis of these sequences showed a strong bias for uridine at the first position (1 T in our data set), in accordance with the preferential binding of PIWI proteins to transcripts starting with U (Fig. 1a). A bias toward adenine at position 10 (a hallmark of secondary piRNAs generated by the ping-pong cycle) was also evident, albeit with a much weaker signal. To study the overlap between the piRNA sequences contained in these databases and other non-coding RNAs, we aligned each sequence against genomic or mitochondria-encoded tRNAs, rRNAs, snRNAs, snoRNAs, YRNAs, and miRNAs. With a zero mismatch allowance, we found 392 (RNAdb 2.0) and 278 (piRBase) piRNAs whose classification as either piRNAs or ncRNA fragments was ambiguous (Table 1 and Supplementary Data 1). These represent 0.23 or 0.85% of the total number of sequences included in each database, respectively. The subset of ambiguous piRNAs shows no preference for 5′ uridine or adenine at position 10 (Fig. 1a). Also, their size distribution is biased toward longer lengths (*P* = 0.008; two-tailed *t-*test), with 26.6% of the sequences being equal to or higher than 30 nt, in contrast to 13.8% in the whole piRNA database (RNAdb 2.0). This is consistent with the slightly longer length of tRNA halves with respect to canonical piRNAs. Altogether, these observations question the classification of this subset as bona fide piRNAs.

**Table 1:** Overlap between annotated piRNAs in piRNA databases (RNAdb 2.0 and piRBase) and non-coding RNAs

Database	# piRNAs	rRNA	tRNA	miRNA	snRNA	snoRNA	YRNA	m_tRNA	m_rRNA	TOTAL	%
MM = 0											
RNAdb 2.0	171,551	111	132	18	5	97	2	13	14	**392**	**0.23**
piRBase	32,826	42	85	13	0	104	8	12	14	**278**	**0.85**
MM = 1											
RNAdb 2.0	171,551	132	478	34	6	114	3	17	17	801	0.47
piRBase	32,826	47	156	19	0	117	15	14	17	385	1.18

*MM* mismatch allowance, *m_tRNA* mitochondrial tRNAs, *m_rRNA* mitochondrial rRNAs

An inspection of the data sources used to create these databases shows that, in contrast to flies and mice, 97% of the unique sequences contained in piRBase are derived from only one study. This study is one of the seminal reports in which piRNAs were first described^5^. In this study, three criteria were used to annotate human piRNAs: (i) cloning and sequencing in human testis, (ii) size in the 25–32 nt range, and (iii) lack of similarity to other known ncRNAs. Thus, piRNA annotation was based on sequencing of a size-selected small RNA library, without direct evidence of PIWI interaction and, as a consequence, human piRNA databases might contain PIWI pathway-independent RNAs. In other model organisms, RIP-seq and CLIP-seq data are available, but lack of highly specific antibodies for the immunoprecipitation of human PIWI proteins has prevented such studies in humans. Nevertheless, contaminating or at least ambiguous piRNAs account for less than 1% of the total number of sequences in the analyzed databases. This might be considered negligible, if it was not for the fact that this subset represents the vast majority of reported non-gonadal piRNAs in humans.

### Reported somatic non-gonadal piRNAs are ncRNA fragments

One of the first reports on mammalian piRNA expression outside of the gonads described a subset of piRNAs expressed in mouse hippocampus^15^. Importantly, co-immunoprecipitation with the murine PIWI protein MIWI was confirmed. In situ hybridization in cultured neurons showed signal from one of these piRNAs in the dendritic compartment, and its antisense suppression suggested a role in dendritic spine morphogenesis. However, we found that all the most abundant piRNAs described in this study were also fragments of YRNAs, C/D box snoRNAs, rRNAs, and even miRNAs (Table 2). One may ask to what extent the biological effects observed upon LNA-based inhibition of the most abundant brain piRNA^15^ (DQ541777; mmu-piR-1889) could be caused by inhibition of full-length RNY1, which is the actual target of such oligonucleotides.

**Table 2:** piRNAs present in the hippocampus of mouse brain are mostly ncRNA fragments

piRNA	Reads	Alternative	Sequence	Reads: MIWI-IP
DQ541777	16,130	RNY1	GGCTGGTCCGAAGGTAGTGAGTTATCTCAA	1
DQ705026	6,257	snoRNA 2	CTGAAATGAAGAGAATACTCTTGCTGATC	0
DQ555094	3,439	rRNA 28S	TGGGGGGCCCAAGTCCTTCTGATCGAGGCCCA	0
DQ719597	2,459	snoRNA 85	GGTCGATGATGAGAGCTTTGTTCTGAGC	0
DQ689086	1,514	snoRNA 27	TGCAATGATGTCATCTTACTACTGAAA	0
DQ540285*	1,433	rRNA 18S	ATCGATGTGGTGCTCCGGAGTTCTCTTCGGGC	0
DQ540981	1,360	rRNA 28S	CGGGCCGCCGGTGAAATACCACTACTCTCA	0
DQ720186	849	miR-3102-3p	AGAGCACCCCATTGGCTACCCAC	0
DQ555093	775	rRNA 28S	TGGGGGGCCCAAGTCCTTCTGATCGAGGC	1
DQ540862	639	snoRNA Z12	CCGGGTGATGCGAATCGTAATCTGAGCCGA	0
DQ540284*	635	rRNA 18S	ATCGATGTGGTGCTCCGGAGTTCTCTTCGGG	0
DQ541506*	580	rRNA 18S	GATCGATGTGGTGCTCCGGAGTTCTCTT	0
DQ539915	304	CDS: MT_CO1	AACATTTCCTGGGCCTTTCAGGAATACCACGA	0
DQ540861	252	snoRNA 104	CCGGGTGATGCGAATCGTAATCTGAGC	0
DQ715526	207	snoRNA 17	CACCAAGATGAGTGGTGCAAATCTGATC	0
DQ543676*	182	rRNA 18S	TCGATGTGGTGCTCCGGAGTTCTCTTCGGGC	0
DQ722288	175	snoRNA D81	TTACTTGATGATAGTAAAAGATCTGATG	0
DQ551351	168	CDS: Fth1	TGCTTCAACAGTGCTTGAACGGAACCCGGT	1
DQ550765	118	snRNA U12	TGCGGGATGCCTGGGTGACGCGATCTGCCCG	0
DQ708131	115	snoRNA 25	TATCTGTGAGGATAAGTAACTCTGAGG	0

The analysis corresponds to every mouse piRNA presented in Table 1 of Lee *et al*.^15^. piRNA accession numbers correspond to the NCBI database. Asterisks denote four piRNAs, which were described as belonging to a large piRNA cluster in chromosome 17 in the referenced report. Our alternative annotation is shown in column 3. Underlined bases correspond to mismatches according to our annotation. Column 5 shows the number of reads corresponding to each piRNA in a Miwi RIP-seq study performed in wild-type adult testis (GEO: GSM822760)

A recent survey of circulating small RNAs present in human blood plasma^18^ revealed a total of 144 piRNAs, many of which were abundant and detectable in every sequenced individual. We took a closer look at these piRNAs, and found that 100% with average expression ≥50 RPM and 68% with a 10 RPM cutoff were in fact ncRNA fragments (Table 3). To better illustrate this point, we divided piRNA reads in RNAdb2.0 (which was the database used in this study) as those starting or not starting with a uridine (148,557 vs. 22,994 sequences, respectively) or those mapping or not mapped to ncRNAs (392 vs. 171,159 sequences, respectively). As a positive control, we analyzed piRNAs in normal human testis^21^. As expected, the distribution of piRNAs in testis matched the distribution of piRNAs in the database (Fig. 1b). This is evident either by using the number of sequences (Fig. 1b, left and middle) or by considering their expression (Fig. 1b, right). In contrast, piRNAs in human plasma were highly enriched in sequences not starting with uridine (ES: enrichment score > 5) and, more strikingly, sequences mapping to ncRNAs (ES > 150 in number of sequences, and ES > 900 when considering relative abundances).

**Table 3:** human plasma piRNAs are mostly ncRNA fragments

piRNA	*N* (%)	RPM (mean)	Alternative	Start/type	Sequence
PIR54042 27	40 (100)	2,295.39	RNAY4	67	CCCCCCACTGCTAAATTTGACTGGCT
PIR2888 30	40 (100)	1,684.62	RNAY1	1	GGCTGGTCCGAAGGTAGTGAGTTATCTCA
PIR58596 31	40 (100)	1,604.31	MT_rRNA 16S	1	GCTAAACCTAGCCCCAAACCCACTCCACCC
PIR43376 32	40 (100)	327.78	MT_tRNA-Val	1	CAGAGTGTAGCTTAACACAAAGCACCCAACT
PIR57581 31	39 (98)	312.29	MT_tRNA-Ser	1	GAGAAAGCTCACAAGAACTGCTAACTCATG
PIR54043 26	40 (100)	59.41	RNAY4	67	CCCCCCACTGCTAAATTTGACTGGT
PIR59288 32	33 (82)	57.53	tRNA-AlaCGC	1	GGGGGGTGTAGCTCAGTGGTAGAGCGCGTGC
PIR40304 32	40 (100)	44.88	MT_tRNA-His	32	TGAATCTGACAACAGAGGCTTACGACCCCTT
PIR41574 31	40 (100)	41.07	piRNA	Cluster Chr5	TGAGATGCGGGAGCTCCGGCGCACACACTC
PIR227919 21	40 (100)	34.69	MT_tRNA-Met	1	AGTAAGGTCAGCTAATTAAG
PIR75448 31	40 (100)	33.04	tRNA-IleAAT	1	GGCCGGTTAGCTCAGTAGGTTAGAGCTTGG
PIR45809 31	34 (85)	28.77	MT_tRNA-Ser	29	TGCCCCCATGTCTAACAACATGGCTTTCTC
PIR57849 32	20 (50)	26.67	CDS: MT_CO2	Antisense	GAGGGCGTGATCATGAAAGGTGATAAGCTCT
PIR57322 27	36 (90)	20.9	CDS: PPP1R3E	Sense, exon	GACAACAACGGCGGCCGTGACTATGC
PIR59786 31	30 (75)	18.06	MT_tRNA-Phe	3′ END	GTTTAGACGGGCTCACATCACCCCATAAAC
PIR37665 28	23 (58)	17.91	piRNA	Cluster Chr11	TCCTGTATTTGCCGAATTGTGGTGTTT
PIR52755 30	35 (88)	17.16	CDS: SPATA31D1	Sense, exon	TGTGCAGAATATTGGTCGAGTTATAAGAG
PIR55478 30	18 (45)	17.05	CDS: C6orf89	Sense, exon	TTCCAGTGCCGAAGACATTGTCAGTCTGT
PIR33872 31	33 (82)	16.74	CDS: VKORC1L1	Sense, exon	TCAAGGCTAAATCTGCTCATGTCGCCACTG
PIR31112 30	34 (85)	15.99	MT_tRNA-Met	3′ END	AAATGTTGGTTATACCCTTCCCGTACTAC
PIR49916 28	31 (78)	15.85	piRNA	Cluster Chr6	TGGGAGTGAAATCAGTGTTTAGGACTA
PIR59752 31	33 (82)	13.49	MT_tRNA-Leu	1	GTTAAGATGGCAGAGCCCGGTAATCGCATA
PIR59421 32	27 (68)	12.25	rRNA_28S	4549	GGTTAGTTTTACCCTACTGATGATGTGTTGT
PIR51124 27	20 (50)	11.73	MT_tRNA-Glu	41	TGGTCGTGGTTGTAGTCCGTGCGAGA
PIR1340 31	33 (82)	10.51	MT_tRNA-Met	5	AGGTCAGCTAATTAAGCTATCGGGCCCATA

Mean abundance (RPM) and number of patients (*N* = 40) in which each piRNA was sequenced were extracted from Freedman *et al*.^18^. The analysis includes every piRNA in the cited study with an abundance ≥ 10 RPM. piRNA accession numbers correspond to the RNAdb 2.0 database. Our alternative annotation is shown in columns 4-5. Underlined bases correspond to mismatches according to our annotation

We still considered the possibility that these ncRNA fragments could be genuine piRNAs secreted to the bloodstream. Of note, the most abundant sequences were fragments of YRNAs (none of them starting with uridine), which could also be detected when analyzing independent plasma sequencing studies (NCBI small read archive: SRR2496797). For instance, the top-ranked plasma piRNA (PIR54042 27; hsa-piR-33043) is a sequence derived from the 3′ end of RNY4 (Table 3). Importantly, sequences mapping to the 5′ end of the same precursor were also detectable in plasma, despite the fact that no piRNAs are annotated in this region (Fig. 1c). This profile is similar in samples from different tissues, but variations in the length and extremes of RNY4 3′ fragments are typically not consistent with PIWI-dependent processing, as they resemble a pattern of exonucleolytic processing from their 5′ end, incompatible with current models of piRNA biogenesis^22, 23^. More probably, fragments of YRNAs in these data sets reflect the most stable and sequencing-prone degradation intermediates of full-length YRNAs, which can be secreted to the extracellular space^24^. Alternatively, 5′ and 3′ fragments from RNY4 could be a result of processing by Dicer, since they correspond to a pair of complementary sequences with protruding ends and a double-stranded core of exactly 21 nucleotides (Supplementary Fig. 1). This reinforces the view that the biogenesis of the piRNAs under study is probably PIWI pathway-independent.

We also analyzed another recent study describing piRNAs in human cancer cells^17^. Here, the authors analyzed transcriptomic data from more than 500 normal tissues and over 5,000 tumor samples from The Cancer Genome Atlas, and discovered 273 and 522 somatic non-malignant and malignant piRNAs, respectively. However, our reanalysis showed that the most abundant piRNAs in cancer cells were either miRNA pathway by-products or ncRNA fragments (Table 4). The top-ranked piRNAs corresponded to miRNAs let-7a-5p and miR-532-5p, which is not expected by chance (*P* = 8 × 10^−9^) since the overlap between piRNA databases and miRBase is rather small (Table 1). The rest of the sequences were mostly fragments of C/D box snoRNAs, mitochondrial tRNAs, and rRNAs. Even though we were surprised to see sequences ≥30 nucleotides corresponding to miRNAs (which are typically 22–23 nt), these showed 100% identity with the pre-miRNAs across the entire sequence length, reinforcing their missclassification as piRNAs.

**Table 4:** The top 20 (most abundant) piRNAs found in human tumors, after analysis of The Cancer Genome Atlas, are miRNAs or ncRNA fragments

piRNA	∑ RPM cancer	Alternative	Sequence
FR072386	38,911,332	miR-let-7a-1	TGAGGTAGTAGGTTGTATAGTTTTAGGGTC
FR182987	3,814,379	miR-532-5p	CATGCCTTGAGTGTAGGACCGT
FR074386	1,093,572	snoRNA 98	ATGCAGTGTGGAACACAATGAACTGAAC
FR140858	1,177,195	miR-106b	TAAAGTGCTGACAGTGCAGATAGTGGTCCTC
FR114004	823,121	snoRNA 1B	TTTCTGTGTGGAATTTGAATATCTGAAA
FR075316	744,575	snoRNA 82	ACCTGATGTTACATTGTAGTGTGCTGATG
FR132879	635,984	snoRNA 58A	CTGCAGTGATGACTTTCTTAGGACACCTTTG
FR064000	407,046	snoRNA 58B	CTGCGATGATGGCATTTCTTAGGACACCTTTG
FR163199	301,387	snoRNA 138	CATGATACTGTAAACGCTTTCTGATG
FR043670	268,790	MT_tRNA-Glu	TGGTCGTGGTTGTAGTCCGTGCGAGAA
FR091055	185,930	miR-744-5p	TGCGGGGCTAGGGCTAACAGCA
FR190827	201,282	snoRNA 6	AGGGGCTGAATGAAAATGGCCTTTCTGAAC
FR090905	168,436	MT_rRNA 16S	GCTAAACCTAGCCCCAAACCCACTCCA
FR016773	172,895	snoRNA 42B	ACTTGTGATGTCTTCAAAGGAACCACTGATG
FR136216	162,693	snoRNA 62A	GGGAGATGAAGAGGACAGTGACTGAGAGAC
FR004819	123,249	snoRNA 114-1	GACGGTGAATACAGGTCTGGAAGTCTGAGGT
FR103462	96,101	snoRNA 98	AAATGCAGTGTGGAACACAATGAACTGAAC
FR089006	95,184	tRNA-Gly	AGCGCCGCTGGTGTAGTGGTATCATGCAAG
FR026913	74,640	snoRNA 89	GAGGAATGATGACAAGAAAAGGCCGAA
FR019019	81,678	snoRNA 107	GTTCATGATGACACAGGACCTTGTCTGAAC

piRNA accession numbers correspond to the Functional RNA database (fRNAdb), as reported by Martinez *et al*.^17^. Our alternative annotation is shown in column 3. Underlined bases correspond to mismatches according to our annotation

## Discussion

The fact that most extragonadal piRNAs in mouse and humans belong to an ambiguous piRNA subset suggests that piRNA expression outside of the gonads is infrequent in mammals. While not excluding the possibility of active piRNA pathways in non-gonadal tissues, detection of somatic bona fide piRNAs might be affected by a subset of highly abundant ncRNA fragments (and even miRNAs), which are reported as piRNAs.

The question is whether the classification of certain ncRNA fragments as piRNAs is erroneous or not. The answer directly impacts on the likelihood of piRNA expression outside of mammalian gonads. The detection of piRNAs circulating in blood plasma^18, 25, 26^ is particularly interesting, and could have an impact on liquid biopsy-based diagnosis. To answer this question, it would be necessary to stress the criteria used for piRNA definition. Accepted properties of piRNAs are their length (24–32 nt), bias toward uridine at 5′, 2′-o-methylation of their 3′ end, and clustering of their coding sequences in the genome^27^. According to their biogenesis, piRNAs can be further classified as genome-encoded primary piRNAs, ping-pong generated secondary piRNAs, and even phased tertiary piRNAs^28^. However, strictly speaking, piRNAs are the small RNAs physically bound and functionally related to PIWI proteins. Thus, a piRNA might not satisfy any of the previous characteristics, but still be a piRNA if capable of specific interaction in the 5′ binding pocket of a PIWI-clade protein. Now the question is, can we affirm that for every sequence deposited in piRNA databases?

At least in humans and mouse, most piRNA databases have grown based on data from the articles which described piRNAs for the first time^29, 30^. Although some of these papers relied on RIP-seq for piRNA identification^7^, others annotated candidate piRNAs based on their sequencing abundance in testis, size in the 25–32 nt range, and lack of similarity to other known ncRNAs^5^. Furthermore, as there is still a lack of suitable antibodies for selective immunoprecipitation of human PIWI proteins^31^, human piRNAs were entirely cataloged from size-selected sequencing of gonad RNA rather than RIP-seq studies. So, the first conclusion is that direct evidence of PIWI interaction is not available for every sequence present in piRNA databases, especially in humans.

**Fig. 2:**
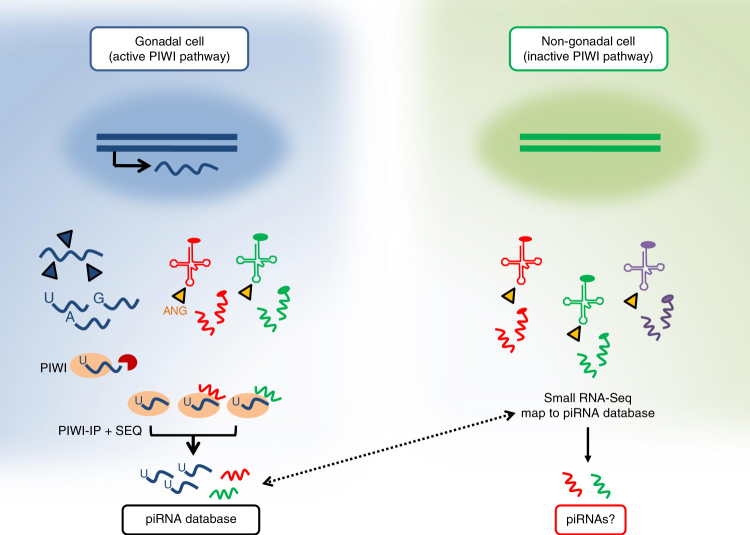
Presence of ncRNA fragments in piRNA databases accounts for piRNA expression in cells where the piRNA pathway is off. A gonadal somatic or germinal cell (blue) produces PIWI pathway-dependent piRNAs (blue RNAs; only the primary piRNA pathway is represented), which are allocated in the 5′ binding pocket of a PIWI-clade protein (orange). ncRNA fragments (red, green) are also present in PIWI immunoprecipitates, but they are not necessarily bona fide piRNAs. When sequencing data are mapped against a piRNA database, ncRNA fragments will be thought as piRNAs, even in the absence of a functional piRNA pathway

Nevertheless, it is still possible that some of the ncRNA fragments present in size-selected small RNA libraries of human testis could be bound to PIWI-clade proteins. If that was the case, they should be regarded as piRNAs. But can be this extrapolated to other tissues? For instance, if a tRNA-derived fragment^32^ interacts with a PIWI protein in the gonads, but is also abundant in a tissue where PIWI proteins are not expressed, would it still be a piRNA in both cases? One disadvantage of using a biogenesis-independent definition of piRNAs is that it makes piRNAs a context-dependent attribute, rather than an intrinsic property of a sequence. Thus, a tRNA fragment should be considered a piRNA if it interacts with PIWI, but the same sequence should not be considered a piRNA in other contexts, when their existence is unrelated to the PIWI pathway (Fig. 2). But this is omitted when mapping somatic small RNA sequencing data to piRNA databases, which actually contain a compendium of small RNAs in the gonads.

The third and more complex issue is that PIWI co-immunoprecipitation should be a necessary but not sufficient condition to claim the presence of bona fide piRNAs. Although we showed that the most abundant piRNAs in mouse hippocampus were ncRNA fragments, we should recognize that the authors did evaluate the presence of these piRNAs in MIWI-IP^15^. Furthermore, tRNA fragments were co-IP with anti-flag antibodies after expressing a flagged version of the human PIWI protein Hiwi2 in a breast cancer somatic cell line^31^. However, this result should be interpreted carefully, as the authors found a very strong correlation (*r* = 0.91) between the tRNA fragments found in Hiwi2-IP and the whole-cell extracts. We would have expected some degree of selection for specific tRNA fragments (such as those starting with uridine, for example).

Relying on PIWI-IP for defining piRNAs can be problematic. In the first place, very specific antibodies are needed. We have found a number of miRNAs after analyzing data from MILI-IP coming from 10 days post-partum (dpp) mouse testis^12^, suggesting a possible contamination with AGO-clade bound RNAs. Secondly, there will usually be a background of RNA fragments stuck to the surface of a PIWI/piRNA complex in any immunoprecipitate, with the contaminants not being truly engaged with the PIWI protein in a biologically meaningful manner. Abundant intracellular RNAs of a similar size (e.g., ncRNA fragments) are risky. By analyzing data from *mili* knockout animals^12^, we have observed that the tRNA fragments that are abundant in MILI-IP do not rely on MILI for neither their biogenesis nor their intracellular stability (Supplementary Fig. 2). Importantly, at 10-dpp MILI is the only PIWI-clade protein expressed in mouse testis^12^, discarding association of tRNA fragments with other PIWI-clade proteins. In contrast, transposable element-targeting piRNAs were decreased as expected in *mili* KO mice. In our opinion, this distinguishes bona fide piRNAs from frequent contaminants in the piRNA size range.

Overall, we have identified that a subset of ncRNA fragments and miRNAs contaminate most human piRNA databases, and that even though the amount of dubious piRNAs is rather low (usually below 1% of the total), this can be problematic when studying somatic piRNA expression. In these types of studies, we strongly encourage a deep analysis of the hits obtained after mapping to a piRNA database, paying particular attention to other possible hits in the genome. We have noted that most of the problematic or ambiguous piRNAs described herein are not included in the piRNA cluster database^33^. This is remarkable, as this database uses small RNA deep-sequencing data as an input, but then uses the genomic coordinates, length distribution, and positional nucleotide composition of mapped reads to define putative piRNA clusters. Thus, many problematic or ambiguous piRNAs are removed when applying more stringent criteria for piRNA definition, such as genomic context and localization. Nevertheless, it should be noted that most “somatic piRNAs” map multiple times in the genome (as a consequence of their sequence identity to tRNAs, rRNAs, and YRNAs) and can show some degree of clustering due to the genomic arrangement of the genes encoding these ncRNAs, or because they map the same ncRNA gene at different positions. An example is the four mouse piRNAs reported to cluster in chromosome 17^15^ (Table 2). Here, the putative cluster is a consequence of the sequences aligning to the same gene (18S rDNA).

It would also be worthwhile to extend these considerations to the miRNA field, although bona fide miRNAs are easier to distinguish based on characteristic sequence patterns, which should correspond to reasonable hairpin precursors. Furthermore, miRBase routinely checks and filters submissions for fragments of rRNAs and tRNAs^34^. Consequently, the overlap between miRBase and ncRNAs was much narrower than in the case of piRNA databases. In a more general view, we would like to argue that solely mapping sequencing data to a given reference (e.g., a piRNA database) should not be considered sufficient proof to claim the expression of a given RNA family, especially when the classification of mapped sequences is ambiguous. In the miRNA field, curated databases with more stringent inclusion criteria (e.g., MirGeneDB) have served to overcome problems arising from the many false positives present in primary repositories^35^. Analogously, the curation of piRNA databases will enable the study of hypothetical piRNA expression outside of mammalian gonads without the interference of piRNA-sized ncRNA fragments.

## Methods

### Bioinformatic analysis

To study the overlap between piRNA databases and ncRNAs, Fasta files containing the complete list of human piRNAs were downloaded from either RNAdb 2.0^29^ or piRBase^36^, and mapped to ad hoc references containing human genomic and mitochondrial rRNAs (downloaded from NCBI), tRNAs (downloaded from the Genomic tRNA Database, GtRNAdb, and the mitochondria tRNA database, mitotRNAdb), small nuclear RNAs, small nucleolar RNAs, YRNAs (all downloaded from NCBI), and miRNAs (downloaded from miRBase). Mapping was performed with the Lastz program contained in the Galaxy Project package, using a seed hit of 19 bp, and returning alignments that covered at least 94 % of the length of each sequence, and showed at least 94 % sequence identity (i.e., a maximum of one mismatch for sequences less than 31 nt). Data from studies reporting piRNAs in human blood^18^ and human testes^21^ were directly extracted from their supplementary Materials section, and the sequences were compared to a reference file containing all piRNAs annotated in RNAdb 2.0 for which we could find 100% identity to ncRNAs. In the case of human piRNAs present in the Cancer Genome Atlas^17^, we computed the number of normalized reads for each putative piRNA across cancer samples, and analyzed the 20 most frequently detected piRNAs in cancer. For piRNAs present in mouse hippocampus^15^, we performed Blast alignments against the NCBI collection of non-redundant nucleotide sequences of mice, and looked for ncRNAs with 100% identity to the putative brain piRNAs.

To analyze the distribution of sequencing reads mapping to human YRNAs in testes and plasma, we extracted Fastq files from the NCBI small Read Archive (SRR2496797) or the European Nucleotide Archive (ERA246774), clipped adaptors, mapped the clipped reads to the human genome (hg19) using Bowtie2, and created a BigWig file from the output, using a bin size of 1, and used the UCSC Genome Browser for visualization. To address the enrichment of putative blood piRNAs in sequences not starting with uridine or matching ncRNAs, we defined a parameter called the Enrichment Score (ES). For the calculation, we divided human piRNAs present in RNAdb 2.0 (reference) in two mutually exclusive categories (e.g., starting [A] or not starting [B] with uridine; identical [A] or not identical [B] to other non-coding RNAs). We then took the 144 piRNAs described in blood plasma^18^ (query), and performed the same categorization. The ES was calculated as the quotient between the number of sequences in the query in categories B and A, divided by the quotient of the number of sequences in the reference in categories B and A. Thus, an ES higher than 1 shows that there is a higher number of sequences in category B in the query than what expected from the distribution in the reference. Alternatively, the sum of the sequencing reads in each category was used, instead of the total number of sequences.

### Data availability

All relevant data not present within the manuscript or supplementary files are available from the authors upon request.

## Electronic supplementary material

Description of Additional Supplementary Files

Supplementary Information

Supplementary Data 1
