# Supplementary material for: Non-coding RNA fragments account for the majority of annotated piRNAs expressed in somatic non-gonadal tissues
Source: Commun Biol. 2018 Jan 22;1:2. doi: 10.1038/s42003-017-0001-7 (PMC6052916; doi:10.1038/s42003-017-0001-7)
Supplement: Supplementary file 1 — Description of Additional Supplementary Files [file 42003_2017_1_MOESM1_ESM.pdf]

File Name: Supplementary Data 1
Description: list of ambiguous human piRNAs present in piRBase (dubious piRNAs). Annotated piRNAs aligning with a maximum of one mismatch to human tRNAs, rRNAs, snoRNAs, YRNAs, miRNAs or mitochondrial tRNAs and rRNAs are shown. Identity: fraction of identical bases between the query and the possible non-piRNA target. Coverage: fraction of the piRNA length included in the alignment block.
