# Supplementary material for: Non-coding RNA fragments account for the majority of annotated piRNAs expressed in somatic non-gonadal tissues
Source: Commun Biol. 2018 Jan 22;1:2. doi: 10.1038/s42003-017-0001-7 (PMC6052916; doi:10.1038/s42003-017-0001-7)
Supplement: Supplementary file 2 — Supplementary Information [file 42003_2017_1_MOESM2_ESM.pdf]

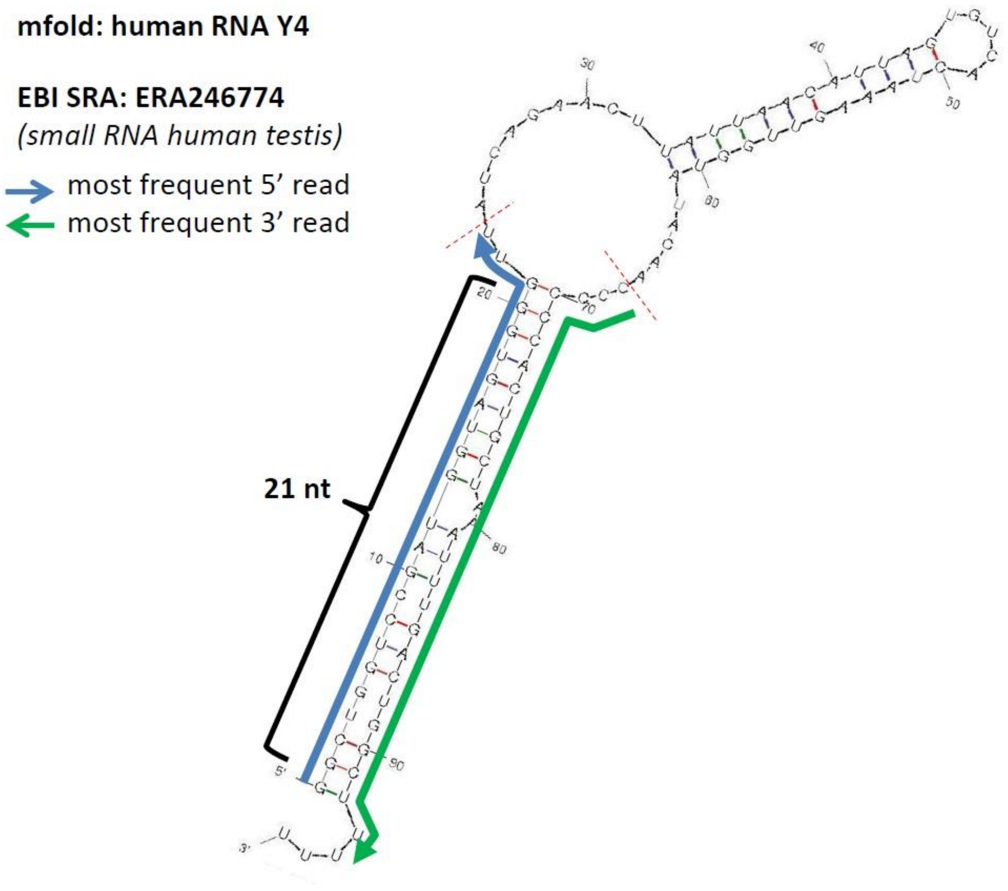

Supplementary Figure 1: prediction of the secondary structure of human RNA Y4 with mfold (unafold.rna.albany.edu/?q=mfold) and schematic representation of the most abundant 5' and 3' reads in human testis (EBI small RNA read archive: ERA246774). The dashed lines show putative cleavage sites consistent with Dicer processing, as the length of the stem measured from the 5' end could fit the enzyme's structural requirements.

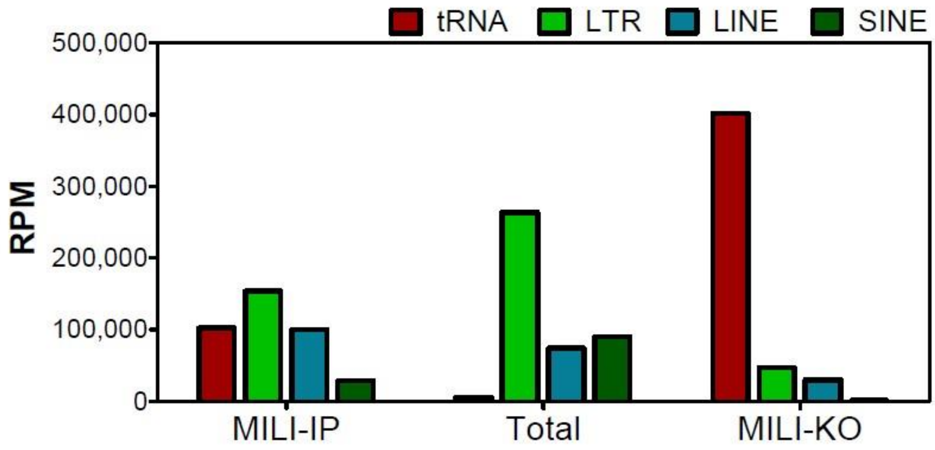

Supplementary Figure 2: Transposon-related piRNAs as dependent on PIWI expression, while tRNA fragments are not. Analysis of small RNA sequencing data from Aravin et al. (2008), corresponding to MILI immunoprecipitation (left), or size-selected total RNA from mouse testes at 10 days post birth (a time point when MILI is the only expressed PIWI protein), either in control (center) or *mili* knock-out animals (right). Bars represent the relative abundance (RPM: reads per million mapped reads) of sequences matching tRNAs (red), or different types of mobile genetic elements (LTR, LINE and SINEs, green)

Supplementary References:

1. Aravin A.A. *et al*. A piRNA pathway primed by individual transposons is linked to de novo DNA methylation in mice. *Mol cell* **31**, 785-99 (2008).
